# Impact of AT2 Receptor Deficiency on Postnatal Cardiovascular Development

**DOI:** 10.1371/journal.pone.0047916

**Published:** 2012-10-29

**Authors:** Daniel Biermann, Andreas Heilmann, Michael Didié, Saskia Schlossarek, Azadeh Wahab, Michael Grimm, Maria Römer, Hermann Reichenspurner, Karim R. Sultan, Anna Steenpass, Süleyman Ergün, Sonia Donzelli, Lucie Carrier, Heimo Ehmke, Wolfram H. Zimmermann, Lutz Hein, Rainer H. Böger, Ralf A. Benndorf

**Affiliations:** 1 Department of Cardiovascular Surgery, University Heart Center, Hamburg, Germany; 2 Institute of Clinical Pharmacology and Toxicology, University Medical Center Hamburg-Eppendorf, Hamburg, Germany; 3 Department of Pharmacology and Heart Research Center Göttingen, Georg-August-University Göttingen, Göttingen, Germany; 4 Institute of Experimental Pharmacology and Toxicology, University Medical Center Hamburg-Eppendorf, Hamburg, Germany; 5 Department of Pharmacology, University of California San Diego, San Diego, California, United States of America; 6 Laboratory of Pharmacology and Toxicology, Hamburg, Germany; 7 Institute of Anatomy and Cell Biology, Julius-Maximilian-Universität Würzburg, Würzburg, Germany; 8 Department of Neurology, University Hospital Hamburg-Eppendorf, Hamburg, Germany; 9 Department of Cellular and Integrative Physiology, University Medical Center Hamburg-Eppendorf, Hamburg, Germany; 10 Institute of Experimental and Clinical Pharmacology and Toxicology, University of Freiburg, Freiburg, Germany; 11 Institute of Pharmacology, Toxicology, and Clinical Pharmacy, Technical University of Braunschweig, Braunschweig, Germany; Brigham & Women's Hospital - Harvard Medical School, United States of America

## Abstract

**Background:**

The angiotensin II receptor subtype 2 (AT2 receptor) is ubiquitously and highly expressed in early postnatal life. However, its role in postnatal cardiac development remained unclear.

**Methodology/Principal Findings:**

Hearts from 1, 7, 14 and 56 days old wild-type (WT) and AT2 receptor-deficient (KO) mice were extracted for histomorphometrical analysis as well as analysis of cardiac signaling and gene expression. Furthermore, heart and body weights of examined animals were recorded and echocardiographic analysis of cardiac function as well as telemetric blood pressure measurements were performed. Moreover, gene expression, sarcomere shortening and calcium transients were examined in ventricular cardiomyocytes isolated from both genotypes. KO mice exhibited an accelerated body weight gain and a reduced heart to body weight ratio as compared to WT mice in the postnatal period. However, in adult KO mice the heart to body weight ratio was significantly increased most likely due to elevated systemic blood pressure. At postnatal day 7 ventricular capillarization index and the density of α-smooth muscle cell actin-positive blood vessels were higher in KO mice as compared to WT mice but normalized during adolescence. Echocardiographic assessment of cardiac systolic function at postnatal day 7 revealed decreased contractility of KO hearts in response to beta-adrenergic stimulation. Moreover, cardiomyocytes from KO mice showed a decreased sarcomere shortening and an increased peak Ca^2+^ transient in response to isoprenaline when stimulated concomitantly with angiotensin II.

**Conclusion:**

The AT2 receptor affects postnatal cardiac growth possibly via reducing body weight gain and systemic blood pressure. Moreover, it moderately attenuates postnatal vascularization of the heart and modulates the beta adrenergic response of the neonatal heart. These AT2 receptor-mediated effects may be implicated in the physiological maturation process of the heart.

## Introduction

Angiotensin II activates at least two heptahelical receptor subtypes, the AT1 and AT2 receptor, and is known to play a major role in the pathophysiology of cardiovascular and renal diseases. In accordance with these findings, pharmacological blockade of either angiotensin II formation by angiotensin conversion enzyme (ACE) inhibitors or angiotensin II-induced activation of AT1 receptors by angiotensin receptor blockers (ARBs) have proven successful strategies for the treatment of hypertension, heart failure, and chronic kidney disease [1]–[4]. Nevertheless, angiotensin II has been reported to also drive multiple physiological effects in the cardiovascular, renal, endocrine, and nervous system [5]. For instance, it is involved in the maturation and growth of the fetal and postnatal heart and kidney [6]–[9]. Indeed, the use of ARBs during pregnancy is associated with an increased probability for cardiac and renal dysplasia in newborn infants, thereby indicating that AT1 receptor blockade and/or unopposed endogenous stimulation of the AT2 receptor, the predominant angiotensin II receptor subtype in the fetal and early postnatal organism, may negatively affect cardiac as well as renal maturation and growth [10], [11]. In this regard, the role of the AT2 receptor in postnatal development of the heart is rather unclear and has not been examined in detail so far. Although the AT2 receptor has been postulated to contribute to pathological cardiac hypertrophy, its role in physiological cardiac hypertrophy and expansion of the coronary blood vessel system remained unclear [12]. Nonetheless, histological analyses indicated that the AT2 receptor is abundantly expressed in cardiomyocytes of the perinatal heart and may also be present in cardiac blood vessels and, therefore, may affect the postnatal growth of cardiomyocytes and cardiac vascular remodeling [13]–[15]. Hence, the aim of this study was to assess the role of the AT2 receptor in postnatal cardiac development by analysing the function, morphology, gene expression, and signal transduction of hearts derived from AT2 receptor-deficient and wild-type mice at different developmental stages.

## Methods

### Animal procedures

Targeted deletion of the murine AT2 receptor gene has been described previously [16]. For this study, AT2 receptor-deficient mice (KO) were used which were backcrossed for more than 10 generations onto the FVB/N background. Hearts from KO as well as wild-type (WT) mice in the postnatal period (1, 7, 14, 56 days after birth) were used for histological, gene expression, and protein phosphorylation analysis. Moreover, protein phosphorylation was analysed in skeletal muscle of mice 7 days after birth.

### Ethics statement

All animal experiments were conducted according to relevant national and international guidelines (German Animal Welfare Act) and were approved by the local Animal Care and Use Committee (Behörde für Soziales, Familie, Gesundheit und Verbraucherschutz - Lebensmittelsicherheit und Veterinärwesen, Hamburg, Germany - 90/06, 53/10, and 74/11).

### Organ removal

Before organ removal, the body weight of the mice was determined. Afterwards, mice were sacrificed and hearts were isolated, weighed, and subsequently ventricular tissue was used for histological analysis or RNA and protein extraction as described below. For histological analysis of cardiac morphology and vessel density, ventricles were fixed in 4% paraformaldehyde (PFA) for 4–6 hours at 4°C. Ventricles were washed in 1× phosphate buffered saline (PBS) and were incubated in 30% sucrose/PBS solution for 12 hours. Lastly, ventricles were frozen in isopentane cooled with liquid nitrogen and stored at −80°C until analysis. Moreover, skeletal muscle biopsies (quadriceps femoris muscle) were collected from 7-day-old WT and KO mice for the analysis of growth-promoting signal transduction pathways.

### Histology and Histomorphometry

For visualization of AT2 receptor expression and distribution in hearts from neonatal mice, formalin-fixed hearts were paraffin-embedded and cut into 4 µm thick sections using a Leica microtom (RM2125RT). Paraffin-embedded sections were stained with an antibody directed against the AT2 receptor (clone AT21-A; 1∶100 dilution; Alpha Diagnostic International, USA). Tissue sections were developed with the Vectastain ABC-AP kit (Vector Laboratories, USA).

For the analysis of cardiac vessel density, transversal sections (10 µm) of cryo-preserved hearts were cut using a cooled (−20°C) cryostat (CTI Cryostat, USA). Capillary density was determined in left ventricular sections with transversely sectioned cardiomyocytes after labelling with Bandeiraea simplicifolia lectin-TRITC (1 µg/ml; Sigma-Aldrich, USA) and counterstained with 4′,6-diamidino-2-phenylindole (DAPI; 1∶1000; Sigma-Aldrich, USA) as previously described [17]. In brief, the number of lectin-positive capillaries and DAPI-stained nuclei were analysed digitally in two visual fields (200× magnification) in ten different sections of each heart using fluorescence microscopy and the Axiovision Measure plus software package (Zeiss, Germany; Figure S1). The capillary index was calculated as the ratio of lectin-positive cells to total number of nuclei (DAPI stain; an indicator of cardiac hypertrophy) per field. In addition, the number of α-SMC-actin-positive blood vessels was analysed digitally in whole cardiac sections stained with an antibody against α-smooth muscle cell actin (1∶400; Sigma-Aldrich, USA), at 25× magnification in ten different sections of the postnatal heart.

### Western Blot analysis

Western blot analysis of phosphorylated S6 ribosomal protein (Ser235/236), Akt (Ser473), and ERK-1/2 (Thr202/Tyr204) was performed as previously described [18], [19] in total cardiac and skeletal muscle extracts from mice 7 days of age using the PathScan® Multiplex Western Cocktail I Detection Kit (Cell Signaling Technology, USA) according to the manufacturer's instructions and normalized to relative elF4E or protein C (1∶1000 dilution) protein content. Moreover, the phosphorylation status p70 S6 Kinase (Thr389, antibody from Cell Signaling Technology, USA) was determined in cardiac tissue. For this purpose, frozen tissue was crushed and lysed using lysis buffer (50 mmol/L Tris/HCl, pH 7.5, 5 mmol/L EDTA, 250 mmol/L NaCl, 0.1% Triton X-100) containing protease inhibitors (0.2 mmol/L phenylmethylsulfonyl fluoride, 1 mg/ml aprotinin, 5 mmol/L dithiothreitol, 1 mol/L Na_3_VO_4_). Equal amounts of cellular proteins (50 µg/lane) were separated by SDS-PAGE and transferred to a nitrocellulose membrane. Afterwards, membranes were incubated with appropriate primary antibody solution according to the manufacturer's instruction. Bound antibodies were detected by appropriate peroxidase-conjugated secondary antibodies and the ECL system (Amersham Bioscience, UK). Densitometric quantification of immunoblots was performed using Gene Tools software. Normalized mean densitometric values obtained in WT tissue were arbitrarily set to 100%.

### Real time RT-PCR

Total RNA from postnatal hearts was isolated as described previously [18] and reverse-transcribed (Superscript II, Invitrogen, USA) by use of random hexamer primers. mRNA expression was quantified using the Applied Biosystems ABI Prism 7900 HT system (TaqMan). We carried out TaqMan reactions in 384-well plates according to the manufacturer's instructions (Applied Biosystems, USA) using pre-made probes for the AT2 receptor (Mm01341373_m1), AT1a receptor (Mm00616371_m1), Atrial natriuretic peptide (ANP; Mm01255748_g1), and Bax (Mm00432051_m1). Glyceraldehyde-3-phosphate dehydrogenase (GAPDH) was used as an endogenous control (probe Mm99999915_g1). We performed relative quantification of gene expression using the standard curve method.

### Isolation of intact adult mouse ventricular cardiomyocytes as well as sarcomere shortening and Ca^2+^ transients measurements

Intact ventricular cardiomyocytes were isolated from wild-type and AT2 receptor knockout mouse hearts (12 weeks old) as previously described [20]. To record intracellular Ca^2+^ transients the isolated cardiomyocytes were loaded with the fluorescent Ca^2+^ chelator Fura-2 (Molecular Probes™, Germany). Thus, sarcomere length shortening and intracellular Ca^2+^ transients of electrically stimulated cardiomyocytes were determined simultaneously with the IonOptix system (IonOptix Corporation, USA). Cardiomyocytes from both genotypes were exposed to angiotensin II (10^−7^ mol/L), isoprenaline (10^−6^ mol/L), alone or in combination with both.

### Preparation, culture, and stimulation of neonatal mouse cardiomyocytes

Isolation of neonatal mouse cardiomyocytes was performed as described previously [21]. After 72 hours of culture, plated cardiomyocytes were starved (0.2% serum) and treated with angiotensin II (10^−7^ mol/L) in the presence or absence of losartan (10^−7^ mol/L) or PD123,319 (10^−7^ mol/L). After 24 hours of incubation, RNA was extracted from the cells.

### Telemetric blood pressure measurements

Radiotelemetric blood pressure measurements were carried out as described [19], [22]. In brief, the mice were anesthetized by intraperitoneal injection of a mixture of ketamine (120 mg/kg) and xylazine (18 mg/kg). A cutaneous incision was made from the submandibular region to the sternum. The left common carotid artery was isolated. The tip of the telemetry device was inserted into the carotid lumen and advanced to the point of lower ligation, which was released to push the catheter forward for a total distance of 10 mm. The telemetric device (TA11PA-C10; Data Sciences International, USA) was placed subcutaneously towards the left abdominal side and fixed with a tissue adhesive. After 10 days, data acquisition started for 1 min every 5 min for 96 hours (Dataquest ART data acquisition, Data Sciences International, USA).

### Echocardiographic measurements

Left ventricular performance and morphology were analysed by echocardiography (40 MHz center frequency single element transducer, Vevo770, Visual Sonics, Canada). Animals were anesthetized with 3% isoflurane, and temperature- and ECG-controlled anaesthesia was maintained with 1.5% isoflurane. Two-dimensional cine loops of a short axis view at mid-level of the papillary muscles were recorded using an ECG-based kilohertz visualization mode. Thicknesses of the anterior myocardial wall (AWTh), the posterior myocardial wall (PWTh), the inner diameter of the left ventricle (LVID) and the area of the left ventricular cavity (Area) were measured in systole (s) and diastole (d), and the fractional shortening (FS) was calculated: FS = (LVID d – LVID s)/LVID d ×100%. For assessment of the effect of beta-adrenergic stimulation on cardiac contractility, echocardiographic parameters were evaluated in 7-day-old KO and age-matched WT mice before and after intraperitoneal application of dobutamine (5 µg/g body weight).

### Ex vivo contractility analyses of ventricular tissue

Hearts from 7-day-old mice were quickly removed from the anesthetized mouse (CO_2_ narcosis) after decapitation. Atrial tissue was removed, left ventricular stripes were cut and were individually mounted vertically from base to apex in a glass tissue bath (25 mL bath volume) and attached to an isometric force transducer (Ingenieurbüro Jäckel, Germany). Hearts were continuously oxygenated (95% O_2_ and 5% CO_2_) at 37°C in modified Tyrode's solution containing 119.8 mM NaCl, 5.4 mM KCl, 1.8 mM CaCl_2_, 1.05 mM MgCl_2_, 0.42 mM NaH_2_PO_4_, 22.6 mM NaHCO_3_, 0.05 mM Na_2_EDTA, 0.5 mM ascorbic acid, 10 mM glucose, and 5 mM pyruvate. Electrically stimulated (rectangular, 1 Hz, 5 ms, 80–100 mA) hearts equilibrated for about 10–45 min and, after exchange of the Tyrode's solution, stretched stepwise to L_max_. Steady state twitch forces were measured under cumulative increases of extracellular calcium (1.8–9 mM) and isoprenaline (0.001–10 µM) in the presence of 1.8 mM calcium. Contraction amplitude data were analysed with BMON2 software (Ingenieurbüro Jäckel, Germany).

### Statistical analyses

Statistical analyses were performed using one-way analysis of variance followed by the Fisher's protected least significant difference test or the student's t-test as appropriate. Variables are expressed as mean±standard deviation (SD) or if indicated as mean±standard error of the mean (SEM). Probability values were considered significant at a *P*<0.05.

## Results

### Influence of AT2 receptor deficiency on postnatal cardiac growth and body weight gain

**Figure 1:**
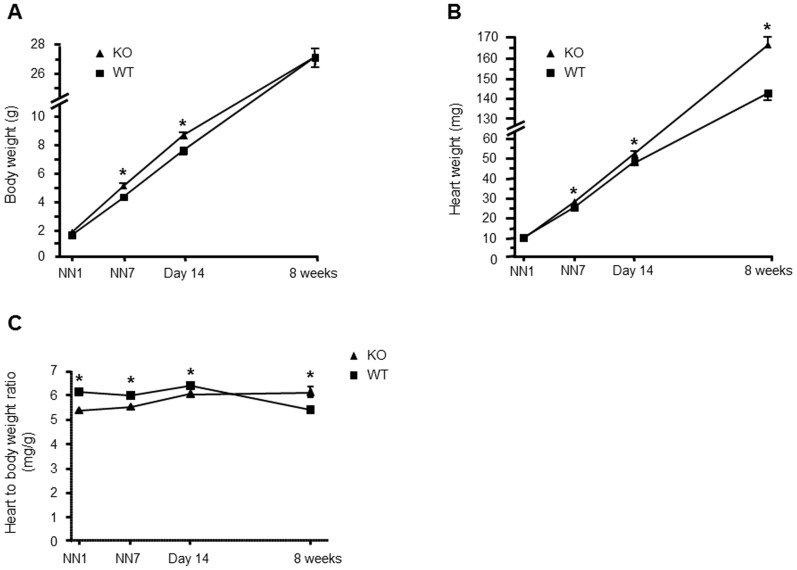
Body weight (A) and heart weight gain (B) as well as heart to body weight ratio (C) of AT2 receptor-deficient (KO) and wild-type (WT) mice during development. Accelerated cardiac and body weight gain in postnatal KO as compared to WT mice resulting in a significantly reduced heart to body weight ratio of neonatal and adolescent KO mice. In contrast, the heart to body weight ratio of adult eight week-old KO mice is significantly increased. * *P*<0.001 vs. WT (n = 43–233/36–210). Data are shown as mean ± SEM.

As shown in Figure 1A, body weight was significantly higher in AT2 receptor-deficient (KO) mice as compared to wild-type (WT) mice at postnatal days 7 (5.1±0.07 g vs. 4.3±0.04 g; *P*<0.001; n = 233/178) and 14 (8.7±0.12 g vs. 7.6±0.10 g; *P*<0.001; n = 186/210), but normalized during adolescence. Accordingly, cardiac growth was also significantly enhanced in KO as compared with WT mice (Figure 1B). Heart weights of 7-day-old (28.1±0.50 mg vs. 25.5±0.29 mg; *P*<0.001; n = 233/178) and 14-day-old KO mice (52.4±0.72 mg vs. 48.2±0.51 mg; *P*<0.001; n = 186/210) were significantly higher than those of age-matched WT mice. However, the heart to body weight ratio of postnatal KO mice was significantly lower than those of age-matched WT mice (Figure 1C). Nevertheless, heart weights of KO mice remained elevated also 8 weeks after birth (165±4.0 mg vs. 142±2.90 mg; *P*<0.001; n = 43/36), whereas the differences in body weight between genotypes disappeared. Accordingly, we observed a higher heart to body weight ratio in 8 week-old KO mice as compared to WT mice (6.0±0.6 mg/g vs. 5.4±0.5 mg/g; *P*<0.001; n = 43/36).

### Analysis of cardiac AT1a and AT2 receptor as well as ANP and Bax mRNA expression

**Figure 2:**
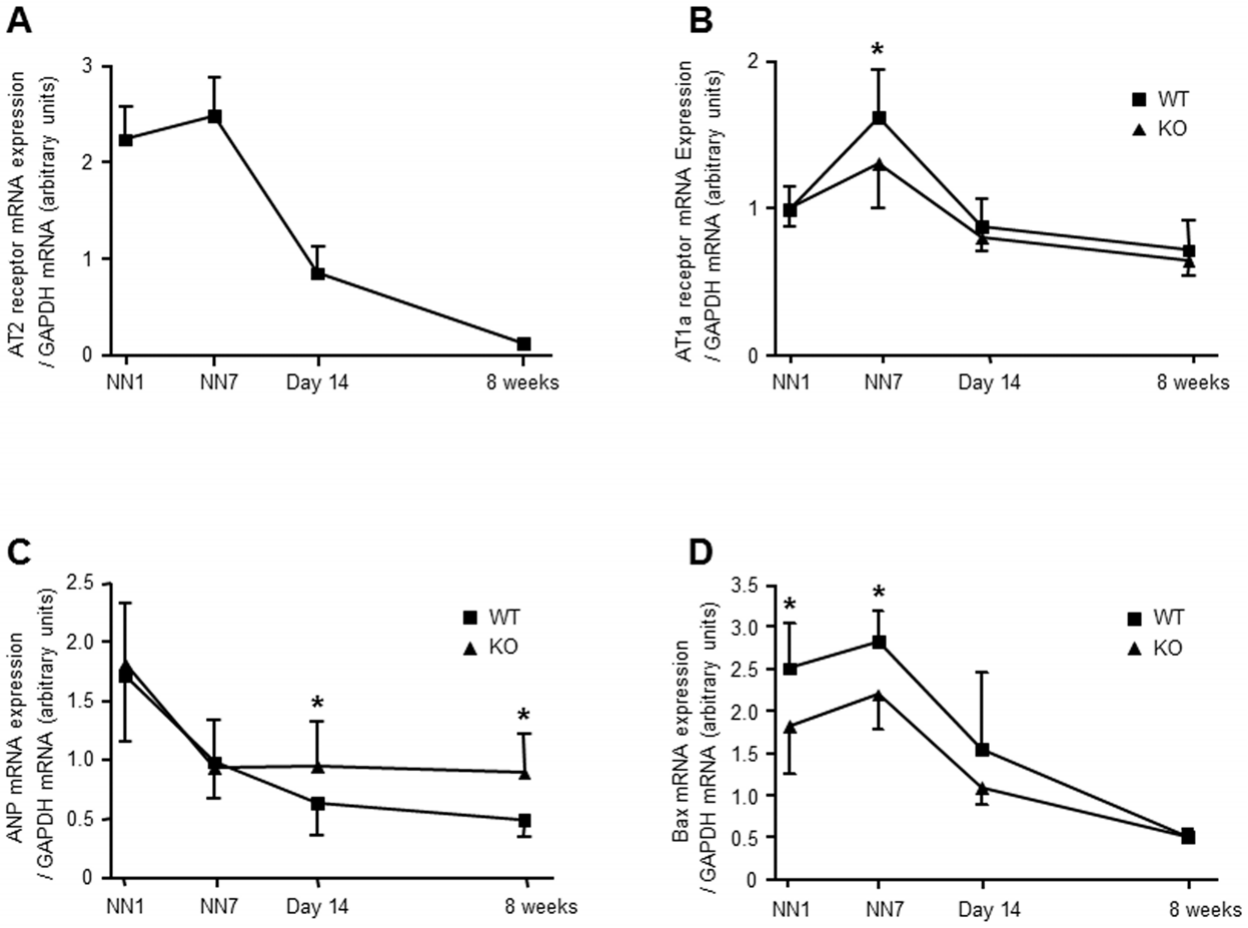
Expression of angiotensin II receptor subtypes (AT1a and AT2), atrial natriuretic peptide (ANP) and Bax in the postnatal heart. A) In WT mice cardiac AT2 receptor mRNA expression peaks at postnatal day 7 (NN7) and declines thereafter. B) Cardiac AT1a receptor mRNA expression also peaked at NN7 and a significantly higher AT1a receptor mRNA expression was then observed in WT mice as compared to that observed in KO mice. C) Cardiac ANP mRNA expression declined in both WT and KO mice during cardiac development in the postnatal period, but ANP mRNA levels remained higher in KO as compared with WT mice from postnatal day 14 on. D) mRNA expression of pro-apoptotic protein Bax is decreased in cardiac tissue of neonatal AT2 receptor knockout mice. * *P*<0.05 vs. WT (n = 10/10). Data are shown as mean ± SD.

In WT mice cardiac AT2 receptor mRNA expression peaked at postnatal day 7 and rapidly declined thereafter (Figure 2A). In addition, immunohistochemical analysis at postnatal day 7 revealed that the AT2 receptor was mainly expressed in endocardium-near cardiomyocytes (Figure S2A). AT1a receptor mRNA expression also peaked at postnatal day 7 in both KO and WT mice, and AT1a receptor mRNA expression was higher in 7-day-old WT as compared to KO mice (1.62±0.32 vs. 1.30±0.30 arbitrary units; *P*<0.05; n = 10/10; Figure 2B) but similar in both genotypes at all other postnatal stages investigated.

Cardiac expression of ANP has been shown to decline during postnatal cardiac maturation and differentiation, whereas it is induced in scenarios of cardiac hypertrophy [23]. Therefore, we investigated cardiac ANP mRNA expression in the postnatal heart (ventricular tissue) of WT and KO mice, respectively. Cardiac ANP mRNA expression declined during neonatal development in both WT and KO mice (Figure 2C). However, ANP mRNA expression remained elevated in KO mice from postnatal day 14 on and was significantly higher as compared to WT mice (day 14: 0.95±0.38 vs. 0.64±0.27 arbitrary units; *P*<0.05; n = 10/10; 8 weeks: 0.89±0.33 vs. 0.49±0.14; *P*<0.01; n = 10/10). The AT2 receptor has been implicated in apoptosis and regulation of the pro-apoptotic protein Bax [24]. Therefore, we investigated the mRNA expression of Bax. Interestingly, mRNA expression levels of Bax were significantly lower in cardiac tissue derived from neonatal KO mice as compared with WT mice but normalized during adolescence (Figure 2D).

### Effect of angiotensin II on ANP mRNA expression in isolated neonatal cardiomyocytes of both genotypes

**Figure 3:**
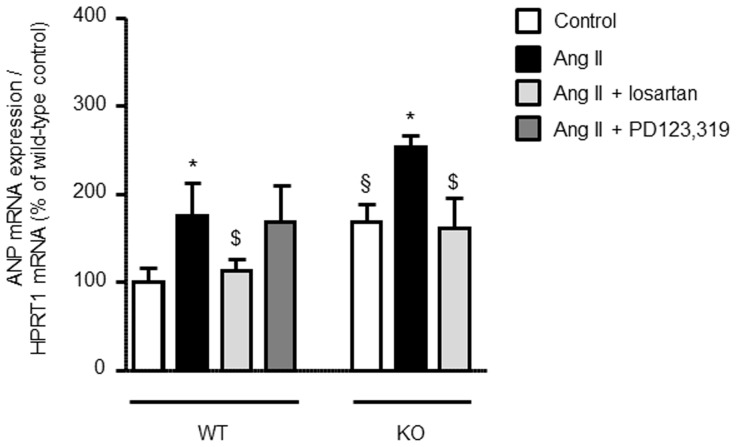
Angiotensin II-induced ANP mRNA expression in neonatal cardiomycytes derived from KO and WT mice. Angiotensin II (Ang II, 10^−7^ mol/L)-stimulated cardiomyocytes from KO mice show a similar increase in ANP mRNA expression as compared to WT mice, but have higher basal ANP mRNA levels. Angiotensin II-induced ANP mRNA expression is significantly reduced by AT1 receptor antagonist losartan, but not by AT2 receptor antagonist PD123,319 (10^−7^ mol/L each). * *P*<0.05 vs. untreated WT or KO cardiomyocytes, respectively, § *P*<0.05 vs. WT control, $ *P*<0.05 vs. Ang II. Data are shown as mean ± SD; n = 6–8.

AT2 receptor-deficient mice showed a reduced heart to body weight ratio in the neonatal and adolescent state, which may be a consequence of direct growth-promoting effects of the AT2 receptor during postnatal cardiac development [25]. To further elucidate this matter, we isolated cardiomyocytes from newborn AT2 receptor knockout and wild-type mice, stimulated the cardiomyocytes with angiotensin II (10^−7^ mol/L) for 24 hours in the presence or absence of the AT1 receptor antagonist losartan (10^−7^ mol/L), and analysed the ANP mRNA expression as a surrogate for cardiomyocyte hypertrophy (Figure 3; values expressed as % of wild-type control). In cardiomyocytes derived from wild-type mice angiotensin II induced an increase in ANP mRNA expression (175±38 vs. 100±21%; *P*<0.05), which was antagonized by the AT1 receptor antagonist losartan (113±13%; *P*<0.05), but not affected by the AT2 receptor antagonist PD123,319 (10^−7^ mol/L; 168±46%). Moreover, neonatal cardiomyocytes derived from AT2 receptor-deficient mice showed a higher basal ANP mRNA expression (169±27%; *P*<0.05 vs. wild-type control), but responded in a similar fashion to angiotensin II as compared with AT2 receptor-expressing wild-type cardiomyocytes (253±14% of wild-type control; *P*<0.05 vs. KO control). Moreover, this increase was again antagonized by losartan (162±38% of wild-type control; *P*<0.05 vs. KO angiotensin II).

### Influence of AT2 receptor deficiency on growth-promoting signal transduction

**Figure 4:**
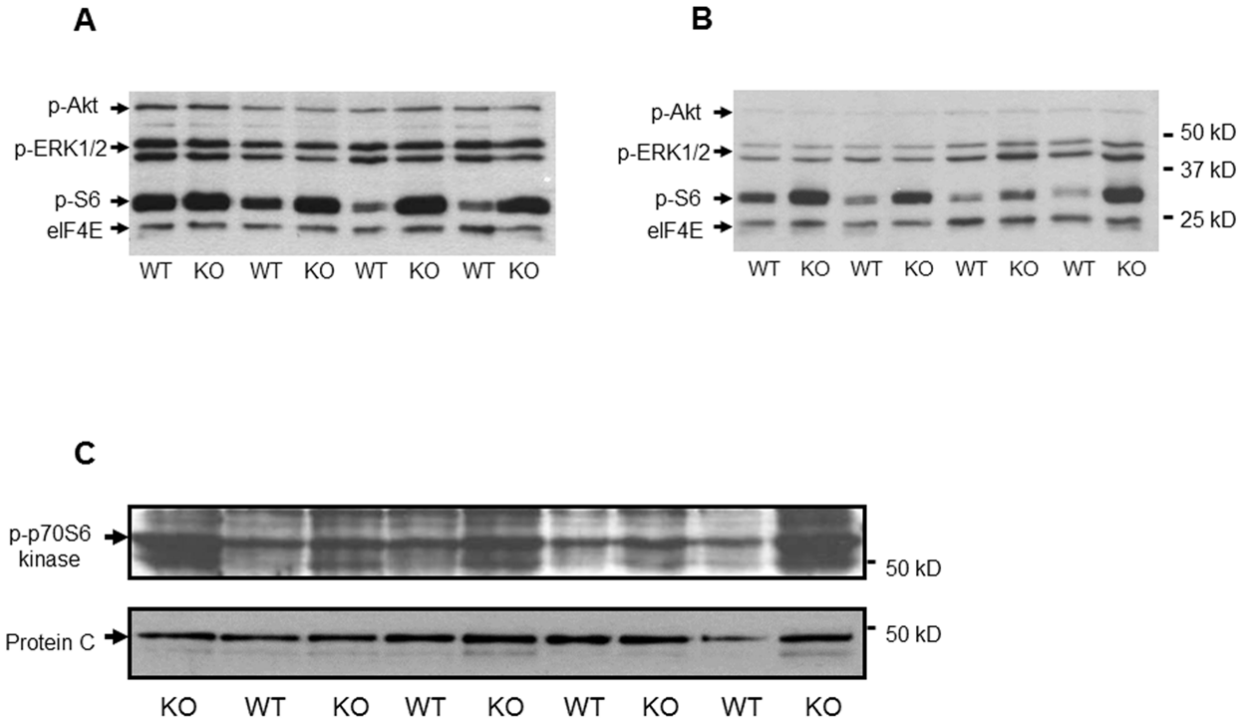
Analysis of growth-promoting signaling transduction pathways in cardiac and skeletal muscle of mice of both genotypes. Phosphorylation of S6 ribosomal protein (Ser235/236) but not of ERK-1/-2 (Thr202/Tyr204) or Akt (Ser473) is significantly increased in cardiac (A) and skeletal (B) muscle of 7-day-old KO as compared to age-matched WT mice. elF4E protein serves as control for protein loading. C) p70S6 kinase phosphorylation status (Thr389) in cardiac tissue derived from 7-day-old KO and WT mice. p70S6 kinase phosphorylation is significantly increased in hearts derived from KO as compared to those derived from WT mice. Protein C serves as protein loading control.

Next, we analysed the influence of AT2 receptor deficiency on important growth-promoting signal transduction pathways in cardiac and skeletal muscle of 7-day-old KO and WT mice. We did not observe significant differences in the phosphorylation status of Akt and ERK-1/-2 in cardiac and skeletal muscle of KO mice, respectively (Figure 4A and 4B). However, phosphorylation of S6 ribosomal protein at Ser235/236 was significantly stronger in KO as compared to WT mice in both cardiac (176.9±58.7% of control vs. 100±36.2%; *P*<0.01; n = 8/8) and skeletal muscle (165.9±35.1% of control vs. 100±30.8%; p<0.001; n = 8/8) tissue. Moreover, phosphorylation of p70S6 kinase (Thr389) was also significantly stronger in cardiac muscle of KO mice as compared with WT mice (167.6±37.5% vs. 100±25.7% of control; *P*<0.01; n = 8/8; Figure 4C).

### Influence of AT2 receptor deficiency on systemic blood pressure

**Figure 5:**
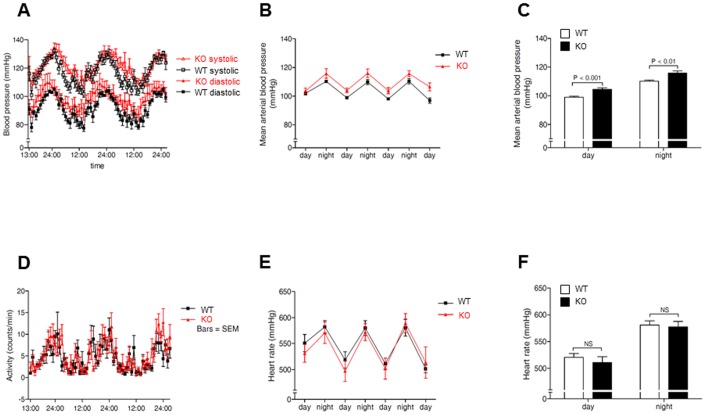
Continuous recording of systolic and diastolic blood pressure in adult AT2 receptor-deficient and wild-type mice (A) and analysis of the average mean blood pressure at daytime or at night (B,C). Measurements show an increased systemic blood pressure in AT2 receptor-deficient mice. Similar activity (D) and heart rates (E,F) were observed in knockout as compared with wild-type mice throughout the observational period. The data are from five wild-type mice and six knockout mice.

In order to analyse the role of systemic blood pressure in cardiac hypertrophy of adult KO mice, we performed telemetric blood pressure measurements in 12-week old AT2 receptor knockout and wild-type mice (Figure 5). We observed a significantly increased mean arterial pressure in KO as compared to WT mice (109.4±4.7 mmHg vs. 103.7±2.0 mmHg, *P*<0.05). In contrast, KO mice did not show an altered physical activity or heart rate (Figure 5D and 5E–F, respectively).

### Influence of AT2 receptor deficiency on cardiac function and ex vivo contractility of ventricular tissue

**Figure 6:**
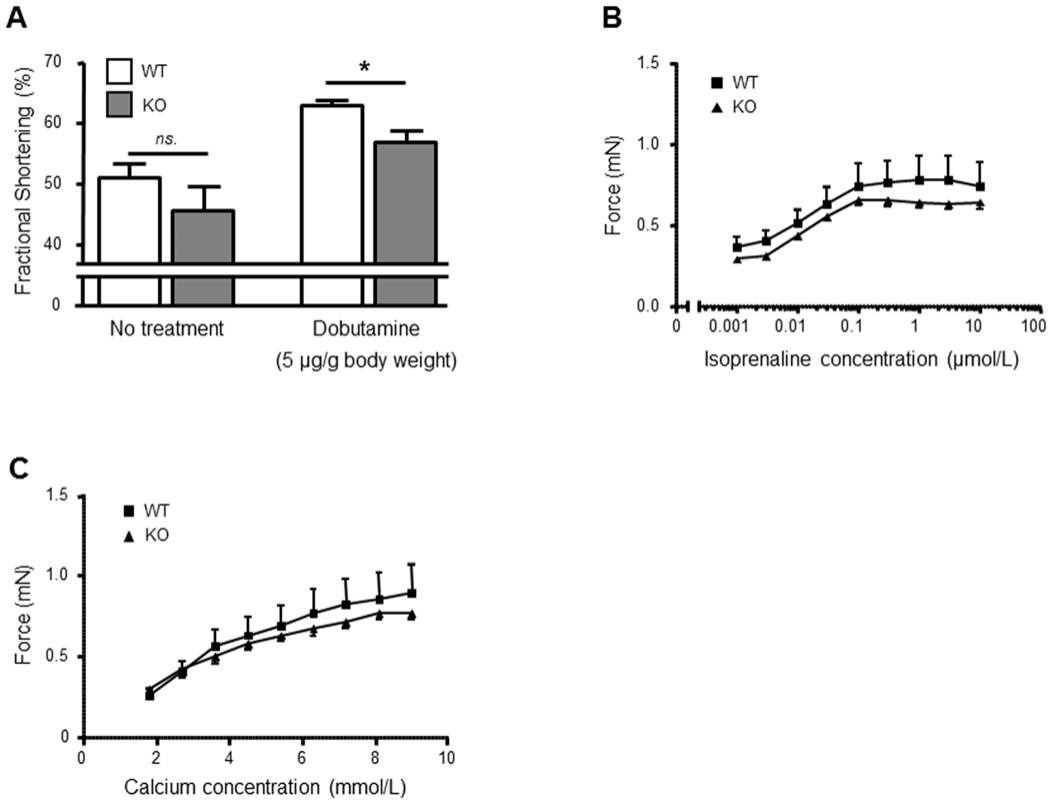
AT2 receptor deficiency is associated with reduced cardiac contractility in the neonatal organism. A) Echocardiographic assessment of cardiac function in 7-day-old KO and WT mice. A significantly reduced fractional shortening (FS) after application of dobutamine (5 µg/g body weight) was observed in KO as compared to WT mice. # *P*<0.01 vs. KO/WT (n = 8/10). Data are shown as mean ± SEM. B) and C) Isoprenaline- and calcium-induced inotropic response of isolated ventricular stripes derived from 7-day-old KO and WT mice (n = 8/6). A non-significant trend towards reduced calcium- and isoprenaline-mediated stimulation of ventricular contractility in KO mice was observed, respectively.

Echocardiographic analyses revealed no significant differences between KO and WT mice at unstimulated conditions. However, after application of dobutamine (5 µg/g body weight), fractional shortening (FS) was significantly lower in KO as compared to WT mice (56.8±5.8% vs. 62.9±3.1%; *P*<0.01; n = 8/10; Figure 6A). To exclude hemodynamic bias, we additionally investigated the ex vivo inotropic response of ventricular tissue derived from both genotypes stimulated with increasing concentrations of isoprenalin and calcium, respectively (Figure 6B and 6C). We did not observe any significant differences between both genotypes: however, a trend towards reduced inotropic response was evident in KO as compared to WT mice.

**Figure 7:**
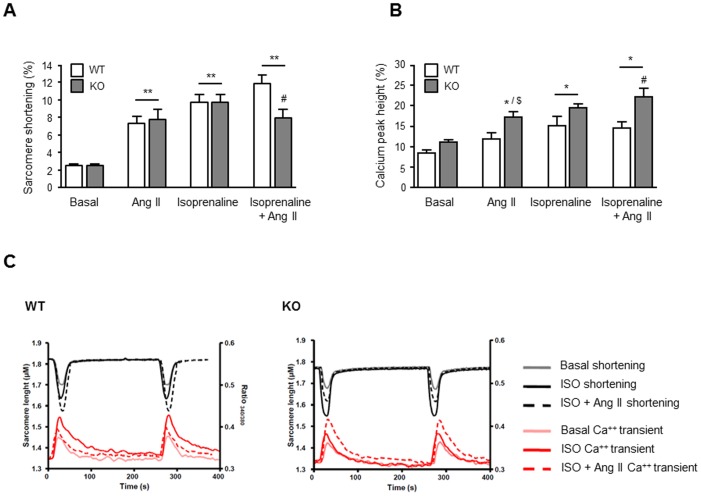
Effects of angiotensin II (Ang II, 10^−7^ mol/L), isoprenaline (ISO, 10^−6^ mol/L), or the combination of both on sarcomere shortening (A) and calcium transients (B) of isolated cardiomyocytes from WT and KO mice (n = 10–36 cardiomyocytes from both three KO and WT hearts were analysed, respectively). ** *P*<0.05 vs. basal sarcomere shortening of WT or KO cardiomyocytes, # *P*<0.05 vs. sarcomere shortening of WT cardiomyocytes treated with isoprenaline in the presence of angiotensin II, * *P*<0.05 vs. basal WT calcium % peak height, $ *P*<0.05 vs. basal KO calcium % peak height, # P<0.05 vs. calcium % peak height of WT cardiomyocytes treated with isoprenaline in the presence of angiotensin II. Data are shown as mean ± SEM. C) Representative recordings of sarcomere shortening (black/grey lines) and calcium transients (pink/red lines) in cardiomyocytes derived from KO and WT mice.

Next, we determined how angiotensin II and isoprenaline influence contractility of cardiomyocytes isolated from WT and KO mice. In the basal conditions (1.25 mM external Ca^2+^), sarcomere shortening did not differ between WT and KO mice (Figure 7A). Exposure to angiotensin II (10^−7^ mol/L) and isoprenaline (10^−6^ mol/L), alone or the combination of both, increased maximal sarcomere shortening in both genotypes. However, KO mice in presence of isoprenaline and angiotensin II showed a significantly reduced sarcomere shortening as compared to WT mice (11.9±3.4% vs. 8.0±3.1%; *P*<0.05). The relative rise in maximal sarcomere shortening was more pronounced than the increase in the Ca^2+^ peak amplitude (Figure 7B). Isoprenaline, and to a minor extent also angiotensin II, increased the calcium peak amplitude similarly in both genotypes. Interestingly, KO mice in presence of isoprenaline and angiotensin II showed a significantly higher calcium peak amplitude compared to WT mice (22.3±6.7% vs. 14.6±5.5%; *P*<0.05). In Figure 7C representative recordings of simultaneously measured sarcomere shortening and Ca^2+^ transients (expressed as F340/380 ratio) are shown of non-stimulated cells and of cells treated with isoprenaline alone, or in combination with angiotensin II.

### Influence of AT2 receptor deficiency on postnatal vascularization of the heart

The AT2 receptor has been implicated in angiogenesis [26], [27] and may hence play a role in the physiological vascularization process of the heart. We observed that at postnatal day 7 the density of α-SMC-actin-positive blood vessels was significantly higher in KO as compared with WT mice (24.1±4.3 vs. 20.7±4.2 blood vessels/mm^2^; *P*<0.05; n = 10/10; Figure S3A). However, during adolescence cardiac density of α-SMC-actin-positive blood vessels normalized in KO mice. In addition, ventricular capillary index was higher in 7-day-old KO as compared to WT mice (1.53±0.19 vs. 1.37±0.15; *P*<0.05; Figure S3B), but again normalized during adolescence.

## Discussion

A potential role of the AT2 receptor in cardiac growth and maturation during fetal and postnatal development has been postulated previously [13], [28]. Indeed, the AT2 receptor appears to be the predominant angiotensin II receptor subtype in the fetal and neonatal heart, whereas its expression level rapidly declines during adolescence [13], [28]. To our knowledge, however, a detailed analysis of AT2 receptor-mediated effects on cardiac development has not been performed so far. In the present study we therefore compared postnatal cardiac development of AT2 receptor-deficient mice to that of age-matched wild-type mice. Interestingly, we observed (1) an accelerated body weight gain, (2) increased heart weights, (3) and a decreased heart to body weight ratio in neonatal and adolescent AT2 receptor-deficient mice. In this regard, increased postnatal body weight gain in AT2 receptor-deficient mice may stimulate concomitant cardiac growth in order to maintain physiological blood supply to the developing organism. However, disproportionate body weight gain and subsequent compensating cardiac growth may also result in a reduced heart to body weight ratio as observed in neonatal and adolescent AT2 receptor knockout mice. It is also possible that the AT2 receptor itself exerts growth-promoting effects on the heart and hence drives physiological cardiac growth in the postnatal period. Indeed, a role of the AT2 receptor in cardiac hypertrophy has been described that may involve the activation and subsequent accumulation of promyelocytic zinc finger protein (PLZF) in the perinuclear region of the cardiomyocyte, thereby inducing the expression of p85a PI3K followed by activation of p70S6 kinase and the development of cellular hypertrophy [25]. In contrast to these findings, we observed considerably increased levels of phosphorylated p70S6 kinase and the downstream target of activated p70S6 kinase, the S6 ribosomal protein, in cardiac tissue of neonatal AT2 receptor knockout mice [Figure 4]. Furthermore, angiotensin II stimulated ANP mRNA expression to a similar extent in neonatal cardiomyocytes isolated from AT2 receptor knockout and wild-type mice and angiotensin II-induced ANP mRNA expression was not reduced by the AT2 receptor antagonist PD123,319 [Figure 3]. Thus, it is possible, but in our opinion rather unlikely, that omission of direct growth-promoting effects of the AT2 receptor is responsible for the decreased heart to body weight ratio in neonatal and adolescent AT2 receptor knockout mice. In contrast to neonatal and adolescent AT2 receptor-deficient mice, 8 week-old AT2 receptor knockout mice had a higher heart to body ratio than wild-type mice, whereas a previous publication showed a reduction of the heart to body weight ratio for six month-old AT2 receptor knockout mice (C57Bl6 background) [29]. Thus, it appears that age and strain background are also important determinants for the relation between heart and body weight. To further elucidate this matter, we performed telemetric blood pressure measurements and observed a significantly increased mean arterial blood pressure in (12 week-old) AT2 receptor knockout mice [Figure 5]. Indeed, an elevated blood pressure has been described previously in AT2 receptor knockout mice [16], [30] and appears to play a major role in angiotensin II-induced cardiac hypertrophy [31]. Hence, it is reasonable that elevated systemic blood pressure contributes to the development of cardiac hypertrophy in 8 week-old AT2 receptor-deficient mice. In this context, not only an inhibitory interplay of AT1 and AT2 receptors expressed in blood vessels may be of importance for the angiotensin II-mediated regulation of systemic blood pressure, but also the antagonistic interaction of both receptors in the central nervous system [28], [32]. Interestingly, the AT2 receptor is abundantly expressed (and often co-expressed with the AT1 receptor) in areas of the hypothalamus and medulla that regulate sympathetic outflow, as well as arterial baroreflex function, and may regulate systemic blood pressure via a reduction of sympathetic tone [32].

Moreover, we observed a considerable increase of phospho-S6 ribosomal protein levels in skeletal muscle of neonatal AT2 receptor-deficient mice [Figure 4B]. These data may point to a growth-inhibitory role of the AT2 receptor in skeletal muscle, which may involve inhibition of the growth-promoting mTOR/p70S6 kinase/S6 ribosomal protein signaling cascade by yet unknown mechanisms. Indeed, p70S6 kinase is a key signaling element of postnatal body growth which regulates S6 ribosomal protein activity [33], [34]. For instance, p70S6 kinase knockout mice display a reduced body weight during postnatal development (approximately −20% as compared to wild-type mice) [33]. Thus, it is possible that AT2 receptor elimination activates the growth-promoting mTOR/p70S6 kinase/S6 ribosomal protein signaling pathway to accelerate body weight gain in postnatal AT2 receptor-deficient mice.

The AT2 receptor has been implicated in the process of angiogenesis [26], [27]. As angiogenesis is a prominent feature during developmental expansion of the cardiac vasculature, we investigated the effect of AT2 receptor deficiency on the density of blood vessels in the developing heart. We observed a moderately higher capillarization as well as a higher density of α-SMC-actin-positive blood vessels in the heart of 7-day-old AT2 receptor-deficient mice as compared to wild-type mice, but vascularization normalized during adolescence. Interestingly, we observed a significantly reduced mRNA expression of the pro-apoptotic protein Bax in cardiac tissue of neonatal AT2 receptor-deficient mice. Thus, the loss of pro-apoptotic effects of the AT2 receptor in knockout mice may contribute to the enhanced vascular proliferation in the heart of these mice observed during the neonatal period [24]. Taken together, these data point to a modest anti-angiogenic effect of the AT2 receptor during cardiac development in vivo. In this context, it has to be mentioned that AT2 receptor expression was high in endocardium-near cardiomyocytes but low in blood vessels of the neonatal mouse heart. Hence, it is not surprising that the effect of AT2 receptor deficiency on postnatal vascularization was rather negligible. In contrast to our observations, Munk and colleagues reported that the AT2 receptor stimulates cardiac angiogenesis in adult mouse cardiac tissue in vitro [26]. This discrepancy may be explained by the experimental setting (in vitro vs. in vivo). Nevertheless, we and others have demonstrated that the AT2 receptor inhibits important steps of the angiogenic process in vitro, such as VEGF-induced endothelial cell migration and tube formation [27], [35]–[38].

To elucidate the role of the AT2 receptor in postnatal cardiac function, we performed echocardiographic analyses in neonatal AT2 receptor-deficient and wild-type mice, and investigated the inotropic response of ventricular tissue isolated from both genotypes. Interestingly, cardiac contractility after dobutamine injection was reduced in AT2 receptor-deficient mice in vivo. Moreover, a trend towards reduced calcium- and isoprenaline-induced inotropic response was evident in ventricular tissue derived from AT2 receptor-deficient mice. In addition, we analysed the effect of isoprenaline (in the presence or absence of angiotensin II) on contraction and calcium transients of isolated adult cardiomyocytes derived from both genotypes. Interestingly, angiotensin II reduced isoprenaline-induced sarcomere shortening in AT2 receptor knockout mice, whereas it rather augmented it in wild-type mice. In contrast, angiotensin II has been described to attenuate isoprenaline-induced contractility in isolated (AT2 receptor-expressing) rat left ventricular papillary muscles [39]. The reason for these discrepant findings is unclear but may be due to the difference in experimental setting (isolated ventricular cardiomyocytes vs. intact ventricular papillary muscles). In addition, angiotensin II further increased isoprenaline-induced calcium peak amplitude in cardiomyocytes derived from AT2 receptor knockout mice, whereas it rather reduced it in wild-type cardiomyocytes. These data suggest that the AT2 receptor may influence cardiac function either by modulating beta-adrenergic signaling and/or calcium handling of cardiomyocytes or indirectly by influencing postnatal maturation of the heart. The data are in line with previously published observations that selective overexpression of the AT2 receptor in ventricular cardiomyocytes results in a higher left ventricular ejection fraction in transgenic mice as compared to wild-type mice [40]. The mechanism responsible for the AT2 receptor-mediated effects on cardiac contractility remains unclear and will have to be determined in future investigations. Nevertheless, the AT2 receptor has been shown to counteract AT1 receptor-mediated activation of myocardial Na^+^/H^+^ exchanger (NHE) which may help to preserve cardiac contractility during physiological and pathophysiological scenarios [41]. Lastly, it has been suggested that the AT2 receptor may also be involved in structural protein accumulation during physiological development of the heart [42] and may stabilize cardiac function by this means.

## Conclusion

AT2 receptor-deficient mice show an accelerated body weight gain, as well as a reduced heart to body weight ratio during postnatal development, a phenomenon possibly attributable to an activation of the growth-promoting p70S6 kinase/S6 ribosomal protein signaling cascade in skeletal muscle of these mice. In addition, deletion of the AT2 receptor leads to an accelerated postnatal vascularization of the heart. Lastly, AT2 receptor deficiency is associated with a reduced cardiac contractility in the neonatal organism. Hence, the AT2 receptor may be implicated in the physiological cardiovascular development of the heart.

## Supporting Information

Figure S1 **Automated detection of blood vessels and morphometrical analyses using the Axiovision Measure plus software package (Zeiss, Germany).** After (immuno)fluorescent staining with an antibody directed against α-SMC-actin (A) or Bandeiraea simplicifolia lectin-TRITC (B) blood vessel density and characteristics were evaluated.
(TIF)

Figure S2 A) Immunohistochemical analysis of AT2 receptor protein expression at postnatal day 7 revealed strong immunostaining in endocardium (Endocard.)-near cardiomyocytes (CM), whereas AT2 receptor protein expression was considerably lower in epicardium (Epicard.)-near cardiomyocytes or in cardiac blood vessels (BV). B) AT2 receptor-deficient heart at postnatal day 7 serving as antibody specificity control.
(TIF)

Figure S3 **Vascular density of hearts derived from postnatal KO and WT mice.** A) and B) Density of α-SMC-actin-positive blood vessels as well as ventricular capillary index is significantly higher in 7-day-old KO as compared with age-matched WT mice. * *P*<0.05 vs. KO/WT (n = 10/10). Data are shown as mean ± SD.
(TIF)
