# Supplementary figures and images for: Impact of AT2 Receptor Deficiency on Postnatal Cardiovascular Development
Source: PLoS One. 2012 Oct 29;7(10):e47916. doi: 10.1371/journal.pone.0047916 (PMC3483305; doi:10.1371/journal.pone.0047916)

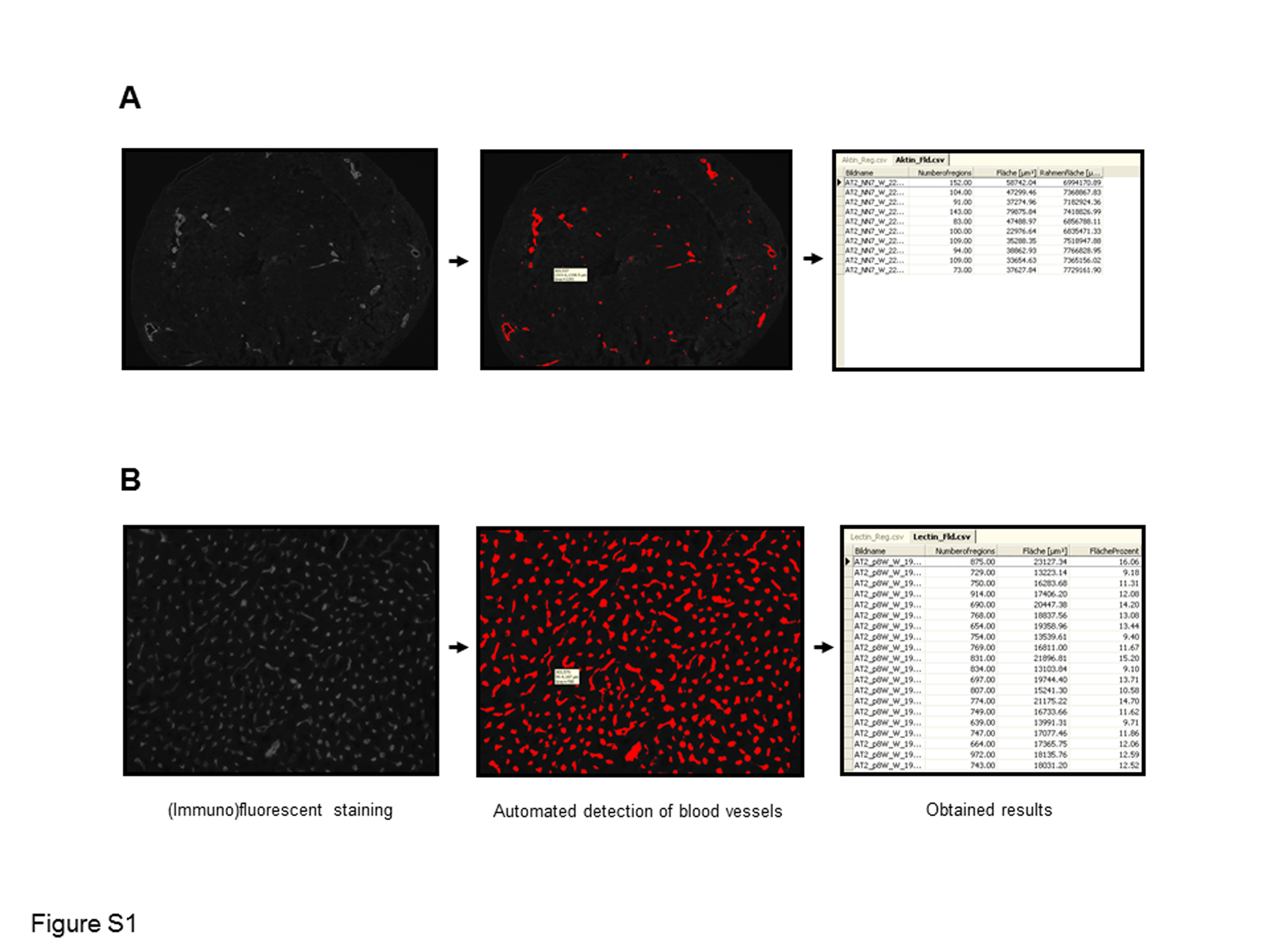

Supplement: Figure S1 — Automated detection of blood vessels and morphometrical analyses using the Axiovision Measure plus software package (Zeiss, Germany). After (immuno)fluorescent staining with an antibody directed against α-SMC-actin (A) or Bandeiraea simplicifolia lectin-TRITC (B) blood vessel density and characteristics were evaluated. (TIF) [file pone.0047916.s001.tif]

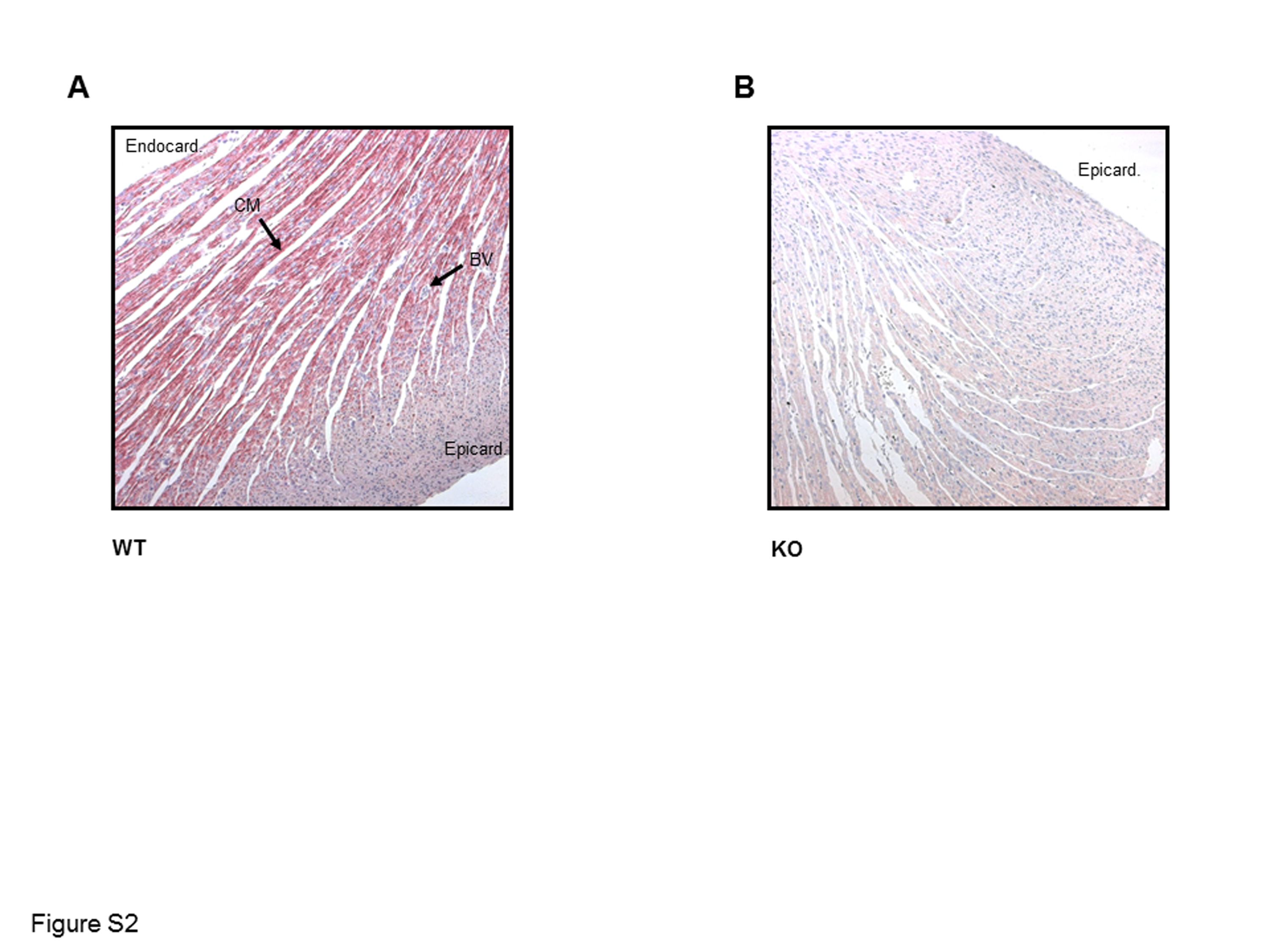

Supplement: Figure S2 — A) Immunohistochemical analysis of AT2 receptor protein expression at postnatal day 7 revealed strong immunostaining in endocardium (Endocard.)-near cardiomyocytes (CM), whereas AT2 receptor protein expression was considerably lower in epicardium (Epicard.)-near cardiomyocytes or in cardiac blood vessels (BV). B) AT2 receptor-deficient heart at postnatal day 7 serving as antibody specificity control. (TIF) [file pone.0047916.s002.tif]
